# Thalamocortical neurons display suppressed burst-firing due to an enhanced I_h_ current in a genetic model of absence epilepsy

**DOI:** 10.1007/s00424-014-1549-4

**Published:** 2014-06-24

**Authors:** Stuart M. Cain, John R. Tyson, Karen L. Jones, Terrance P. Snutch

**Affiliations:** Michael Smith Laboratories and Djavad Mowafaghian Centre for Brain Health, University of British Columbia, 219-2185 East Mall, Vancouver, BC Canada V6T 1Z4

**Keywords:** Absence, Seizure, Burst-firing, Epilepsy, I_h_, HCN

## Abstract

Burst-firing in distinct subsets of thalamic relay (TR) neurons is thought to be a key requirement for the propagation of absence seizures. However, in the well-regarded Genetic Absence Epilepsy Rats from Strasbourg (GAERS) model as yet there has been no link described between burst-firing in TR neurons and spike-and-wave discharges (SWDs). GAERS ventrobasal (VB) neurons are a specific subset of TR neurons that do not normally display burst-firing during absence seizures in the GAERS model, and here, we assessed the underlying relationship of VB burst-firing with I_h_ and T-type calcium currents between GAERS and non-epileptic control (NEC) animals. In response to 200-ms hyperpolarizing current injections, adult epileptic but not pre-epileptic GAERS VB neurons displayed suppressed burst-firing compared to NEC. In response to longer duration 1,000-ms hyperpolarizing current injections, both pre-epileptic and epileptic GAERS VB neurons required significantly more hyperpolarizing current injection to burst-fire than those of NEC animals. The current density of the Hyperpolarization and Cyclic Nucleotide-activated (HCN) current (I_h_) was found to be increased in GAERS VB neurons, and the blockade of I_h_ relieved the suppressed burst-firing in both pre-epileptic P15–P20 and adult animals. In support, levels of HCN-1 and HCN-3 isoform channel proteins were increased in GAERS VB thalamic tissue. T-type calcium channel whole-cell currents were found to be decreased in P7–P9 GAERS VB neurons, and also noted was a decrease in Ca_V_3.1 mRNA and protein levels in adults. Z944, a potent T-type calcium channel blocker with anti-epileptic properties, completely abolished hyperpolarization-induced VB burst-firing in both NEC and GAERS VB neurons.

## Introduction

Burst-firing of neurons in the thalamocortical system is a characteristic feature accompanying the spike-and-wave discharges (SWDs) observed on electroencephalography (EEG) recordings during absence seizures, as well as being associated with other types of generalized and partial-onset epilepsies [4, 14]. The thalamocortical system is comprised of three key loci: glutamatergic corticothalamic pyramidal neurons (CPNs) in layers V/VI of the cortex which project to both the reticular thalamic nucleus (RTN) and to thalamic relay (TR) neurons, glutamatergic TR neurons which project to CPN and RTN neurons, and GABAergic RTN neurons which project to TR neurons and also synapse onto other neurons within the RTN. During absence seizures it is generally believed that CPN and TR neurons become locked in a self-propagating oscillatory loop, at least in part due to the hyperpolarizing rhythmic and synchronizing drive provided by burst-firing TR neurons. Simultaneous EEG and intracellular recordings have defined TR regions that burst-fire in a phase-locked manner with SWDs in some animal models of absence seizures [19, 21, 41], which suggests that these regions are critical in the generation and/or propagation of seizure activity. Enhanced low-threshold T-type calcium currents can contribute towards the hyperexcitable burst-firing of TR neurons and are thought to induce or aid the propagation of absence seizures [7, 18]. In support, knockdown of the Ca_V_3.1 T-type calcium channel expressed in TR neurons has been shown to protect against pharmacologically induced absence seizures [26, 43].

The Genetic Absence Epilepsy Rats from Strasbourg (GAERS) model of absence epilepsy displays spontaneous seizures with a cortical focus and concomitant strong contributing influence of the RTN [33, 36, 37, 42, 51]. This model develops absence seizures spontaneously wherein all animals from the inbred strain exhibit seizures by around 3 months of age. To date, two genetic alterations have been discovered in the GAERS genome with one of these, a missense mutation in the Ca_V_3.2 T-type calcium channel inducing a gain-of-function effect through an enhanced rate of recovery from inactivation [20, 38]. While GAERS is a well-studied model of absence epilepsy, it remains to be described whether TR neurons in GAERS display SWD phase-locked burst-firing during absence seizures. A number of studies have performed intracellular recordings in first- and second-order TR neurons, however as yet none have reported robust oscillatory burst-firing during absence seizures in GAERS [9, 31–35, 41]. Conversely, second-order TR neurons in the WAG/Rij model of absence epilepsy have been shown to burst-fire in synchrony with SWDs [19]. First-order subsets of TR neurons include those in the ventrobasal (VB) and ventromedial (VM) thalamic regions, which overwhelmingly project to, and receive input from, the somatosensory cortex and motor/premotor frontal cortex [12, 15, 24, 25, 30]. The VB and VM regions are of particular interest since the interconnected cortical loci display SWD electrocorticogram (ECoG) activity during absence seizures and are at, or close to the location of originating seizure focus [36, 51]. Interestingly, simultaneous EEG and intracellular recordings of VB and VM TR neurons demonstrate that neither region generates rhythmic burst-firing during SWDs, despite having the ability to do so, but instead displays a sporadic action potential-generating, sub-threshold membrane oscillation [9, 31, 33]. In order to further investigate the underlying mechanism of this suppressed ability to burst during SWDs, the burst-firing properties of VB neurons was assessed in non-epileptic control (NEC) and both pre-epileptic and epileptic GAERS animals. We further examined VB properties with respect to I_h_ and T-type calcium channels at the whole-cell current, protein, and mRNA expression levels.

## Materials and methods

### Acute thalamic slice patch-clamp recordings

NEC and GAERS rats (male and female; bred by the Zoology Department and Animal Resource Unit at The University of British Columbia, Canada) P7–P9 (T-type currents, voltage clamp), P15–P20 (neonatal current clamp, I_h_ voltage clamp), and P120–P150 (adult current clamp, I_h_ voltage clamp) were used in acute brain slice patch-clamp experiments. P15–P20 rats were briefly anesthetized using isoflurane and sacrificed by cervical dislocation, and the brains were rapidly removed. Adult rats were anesthetized using inactin (80 mg/kg i.p) and intracardiacally perfused with ice-cold sucrose solution containing in mM: 234 sucrose, 24 NaHCO_3_, 1.25 NaH_2_PO4, 11 glucose, 2.5 KCl, 0.5 CaCl_2_, and 6 MgCl_2_, bubbled with 95 % O_2_:5 % CO_2_ for 15 min prior to decapitation. Brain tissue was glued to a cutting chamber, which was then filled with ice-cold sucrose solution. Horizontal brain slices containing the whole thalamus (neonate ∼300–350 μm thick; adult ∼200 μm thick) were cut from the level of the ventral RTN/VB and incubated for a minimum of 1 h at 34 °C in a current-clamp recording solution containing in mM: 126 NaCl, 2.5 KCl, 26 NaHCO_3_, 1.25 NaH_2_PO4, 2 CaCl_2_, 2 MgCl_2_, 10 glucose, 1 kynurenic acid, and 0.1 picrotoxin, bubbled with 95 % O_2_:5 % CO_2_. Slices were transferred to a recording chamber superfused with either current-clamp recording solution or voltage-clamp recording solution and maintained at 33–35 °C. Voltage-clamp recording solution contained (in mM): 126 tetraethylammonium-Cl, 2.5 KCl, 26 NaHCO_3_, 1.25 NaH_2_PO4, 2 CaCl_2_, 2 MgCl_2_, 10 glucose, 1 kynurenic acid, and 0.1 picrotoxin, bubbled with 95 % O_2_:5 % CO_2_. VB neurons were visualized using a DIC microscope (Axioskop 2-FS Plus, Carl Zeiss) and infrared camera (IR-1000, DAGE MTI) and visually identified by their location, morphology, and orientation. All recordings were undertaken using a Multiclamp 700B amplifier and pClamp software version 9 (Molecular Devices). The recording chamber was grounded with a Ag/AgCl pellet.

P7–P9 animals were used in voltage-clamp experiments to utilize animals that were as old as possible, while minimizing penalties associated with space clamp of dendritic T-type calcium channels. Calcium currents from VB neurons displaying poor clamping properties as determined by slow kinetics or gap junction-mediated depolarization of adjacent neurons as determined by “double-peak” currents were discarded. Whole-cell voltage-clamp recordings were undertaken using fire-polished borosilicate glass pipettes (3–5 MΩ) filled with an intracellular of composition containing in mM: 120 Cs-methanesulfonate, 10 4-(2-hydroxyethyl)-1-piperazineethanesulfonic acid (HEPES), 0.5 MgCl_2_, 1 CaCl_2_, 10 tetraethylammonium-Cl, 5 4-aminopyridine, 11 ethylene glycol tetraacetic acid (EGTA), 4 MgATP, and 0.5 NaGTP, pH adjusted to 7.2 using CsOH and osmolarity adjusted to 290 mOsm/kg using D-mannitol. The liquid junction potential for voltage-clamp solutions was calculated as +12.2 mV and corrected online using the Multiclamp interface. Series resistance was monitored regularly during recordings for variability, and data from neurons displaying access resistance of greater than 24 MΩ were discarded. Series resistance was compensated by 70 % correction. Currents recorded under voltage-clamp conditions were sampled at 20 kHz and filtered at 2.4 kHz and leak corrected off-line. The current–voltage (I–V) relationship was obtained by depolarizing the membrane with 200-ms pulses from a holding potential of −90 mV. Test pulses from −70 to +10 mV were applied at 5-mV steps. In a separate protocol for assessing VB neuron calcium currents, a 100-ms pre-pulse to −50 mV was applied during the I–V protocol to inactivate the T-type component, allowing the high-voltage-activated calcium currents to remain. These were then subtracted from calcium current recorded in the absence of a pre-pulse to acquire the T-type component. Currents recorded during the I–V protocol were normalized to whole-cell capacitance to yield the current density. Whole-cell capacitance was calculated using a 5-mV step from −90 to −85 mV for 40 ms. The integral of the transient was taken and divided by 5 mV (the step magnitude) to yield the whole-cell capacitance.

Hyperpolarization-activated current was elicited by applying a 1,000-ms pre-pulse at membrane potentials from −110 to −60 mV (5-mV increments; see Fig. 3a). Tail currents were then induced via a step to −110 mV for 200 ms. The protocol was repeated in the presence of ZD7288 (20 μM) leaving the leak current remaining, which was then subtracted from the control current to isolate the ZD7288-sensitive component. Maximum tail currents were used for analysis.

Whole-cell current-clamp recordings were undertaken using fire-polished borosilicate glass pipettes (4–6 MΩ) filled with the following solution containing in mM: 120 K-gluconate, 10 HEPES, 1 MgCl_2_, 1 CaCl_2_, 11 KCl, 11 EGTA, 4 MgATP, and 0.5 NaGTP, pH adjusted to 7.2 using KOH and osmolarity adjusted to 290 mOsm/kg using D-mannitol. The liquid junction potential for current-clamp solutions was calculated as +13.3 mV and corrected off-line. To evaluate basic neuronal responses to hyperpolarization and depolarization, DC current was injected from −110 to +200 pA in 10-pA increments for a duration of 1,000 ms at the cell’s intrinsic resting membrane potential. Neurons that did not exhibit burst-firing (as determined by a minimum of three action potentials within 100 ms during the current step) in response to depolarizing current steps were discarded. Membrane potential responses under current-clamp conditions were sampled at 50 kHz and filtered at 10 kHz. Bridge balance was monitored during recordings, and any neurons displaying bridge balance values greater than 20 MΩ were discarded. Capacitance neutralization was performed between 3.8 and 4.2 pF.

Burst inflection and deflection points were measured by integrating the burst-firing voltage trace and determining the time point at which the neuron began to exponentially depolarize and repolarize, respectively. The corresponding membrane potential to this time point was recoded.

Data analysis was performed using Clampfit version 9 and Origin version 8.6. Data followed a normal distribution, and statistical significance was calculated using Student’s two-sample *t* test (paired where relevant). Data are plotted as mean ± standard error.

### Drugs

Drugs were dissolved in distilled H_2_0 (ZD7288) or dimethylsulfoxide (DMSO; Z944) and used at a minimum 1:1,000 dilution.

### Protein expression using Western blotting

VB thalamic tissue was dissected from 500-uM-thick horizontal brain slices of six male and female individual P120–P150 NEC and GAERS animals. Brain slices were cut in ice-cold sucrose solution using a vibratome as described above. Thalamic extracts were homogenized using Laemmli buffer and liquid nitrogen. Hyperpolarization and Cyclic Nucleotide-activated (HCN)-1 samples were loaded with 10 μg per lane. HCN-2, HCN-3, and HCN-4 samples were loaded with 20 μg per lane. Sample lysate concentration was determined using the Pierce 660 nm Protein Assay (Fisher # PI22660) with the addition of the IDCR reagent (Fisher # PI22663) to compensate for the use of Laemmli buffer in the sample preparation. All samples were run on NuPAGE^®^ Novex^®^ 4–12 % Bis-Tris Midi Gels (Invitrogen) using MOPS Buffer. The samples were blotted overnight at 4 °C, running at 30 V. The transfer buffer contained (in mM) Tris–HCl (25) and glycine (192), 20 % methanol and 0.1 % SDS. Samples were blotted on to nitrocellulose membrane (Amersham Hybond ECL 0.45 um, Fisher # 45000929). After transfer, the membranes were equilibrated for 10 min in Tris-buffered saline (TBS) + 0.1 % Tween-20, then blocked for 1 h at room temperature in TBS-T plus 2 % non-fat milk powder. The primary antibodies were diluted in TBS-T plus 2 % milk and incubated for 2 h at room temperature. The membranes were washed three times for 20 min in 2 % milk buffer, followed by incubation for 1 h at room temperature with the secondary antibodies diluted in 2 % milk buffer. After the secondary incubation, the membranes were washed three times for 10 min in TBS plus 0.05 % Tween-20. The ECL reagent was then added, and the membranes exposed to Amersham Hyperfilm ECL (Fisher # 45001505). Antibodies: anti-HCN-1 rat ascites at 1/4,000 (Millipore # MAB5594), anti-HCN-2 rat ascites at 1/2,000 (Millipore # MAB5596), anti-HCN-3 rat ascites at 1/2,000 (Fisher # MA3-902), anti-HCN-4 rat ascites at 1/2,000 (Fisher # MA3-903), anti-vinculin mouse monoclonal at 1/10,000 (Sigma # V9131), secondary goat anti-rat HRP at 1/5,000 (Santa Cruz #sc-2065), and secondary goat anti-mouse poly-HRP at 1/10,000 (Fisher # PI32230), anti-Ca_V_3.1 rat at 1/3000 (Alomone #ACC-021) and secondary goat anti-rabbit poly-HRP at 1/5000 (Fisher #PI32260). The densitometric results were analyzed using Image Studio Lite software from LI-COR.

### mRNA expression analysis using quantitative real-time PCR (qPCR)

VB thalamic tissue was dissected from 500-uM-thick horizontal brain slices of three individual P120–P150 NEC and GAERS animals followed by total RNA extraction. Brain slices were cut in ice-cold sucrose solution using a vibratome as described above. Each VB thalamic sample was homogenized in a sterile glass-Teflon homogenizer in the presence of TRI-Reagent (Ambion) and total RNA isolated using a MagMAX^TM^-96 for Microarrays preparation kit (Ambion). Thalamic tissue from P9 animals was acquired using the same method, although isolation of the VB thalamic tissue was not as accurate due to the size of the sample. Total complementary (c)DNA was then synthesized from 2 μg of total RNA using a High Capacity cDNA Reverse Transcription kit (Applied Biosystems). Real-time PCR reactions were performed using Applied Biosystems reagents and TaqMan probes to the respective gene targets on an Applied Biosystems 7500 system. Primer assays were used for the detection of Ca_V_3.1 (Life Technologies; Rn00581051_m1), Ca_V_3.2 (Life Technologies; Rn01460351_g1), or Ca_V_3.3 (Life Technologies; Rn01505215_m1) mRNA. A rat actin B mRNA assay (Life Technologies; 4352340E) was run in parallel with probes as a control for total cDNA input to allow for comparison. Amounts of each isoform in the sample were calculated and scaled using relative actin B amounts before being compared. Target and control probe reactions were run in triplicate and averaged for each sample.

## Results

### Rebound bursts in VB neurons responding to short duration hyperpolarization are selectively suppressed in adult GAERS

**Fig. 1:**
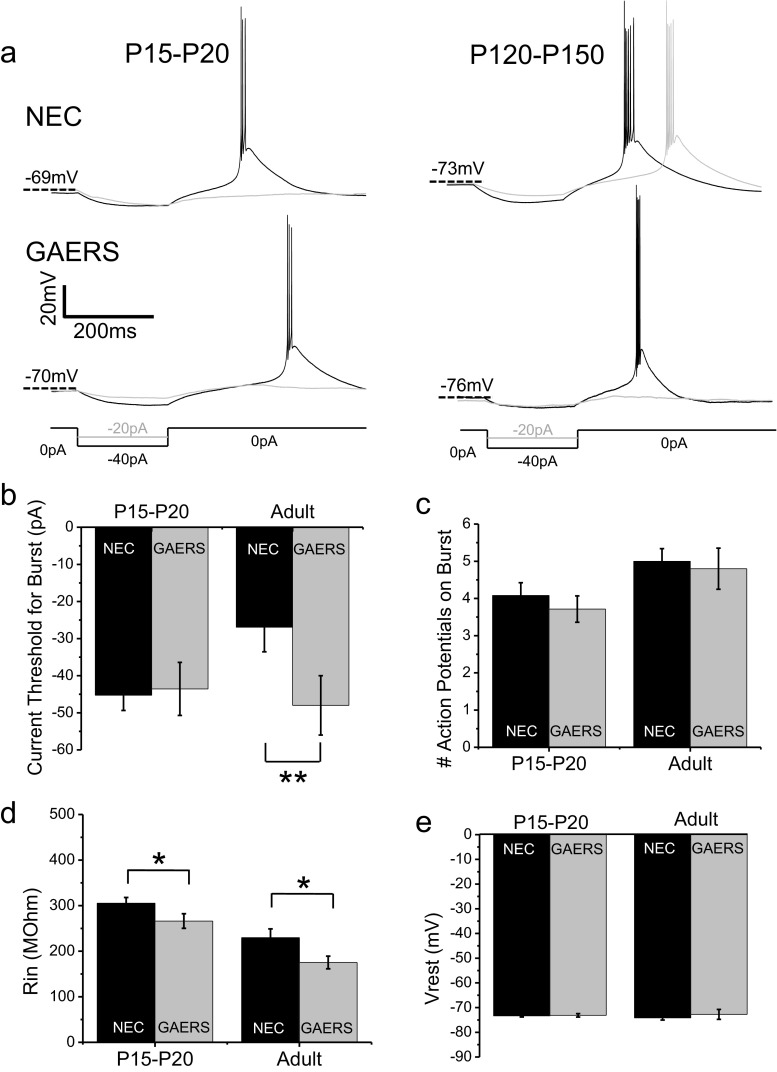
GAERS VB neurons display suppressed burst-firing that is dependent on age. Representative current-clamp recordings from P15–P20 (**a**, *left panels*) and adult (**a**, *right panels*) NEC (**a**, *upper panels*) and GAERS (**a**, *lower panels*) VB neurons in response to a 200-ms hyperpolarizing current injection. Note that adult VB neurons require a greater magnitude current to burst-fire, whereas P15–P20 neurons do not. Mean data for 200-ms current injection required for threshold burst-firing for P15–P20 (NEC (*n* = 38; *black columns*), GAERS (*n* = 14; *grey columns*)) and adult (NEC (*n* = 13; *black columns*), GAERS (*n* = 10; *grey columns*)) VB neurons is shown in (**b**). The number of action potentials per burst at threshold is shown in (**c**). Mean data for input resistance is shown in (**d**). Membrane potential at rest is displayed in (**e**). *p < 0.05, **p < 0.01

**Fig. 2:**
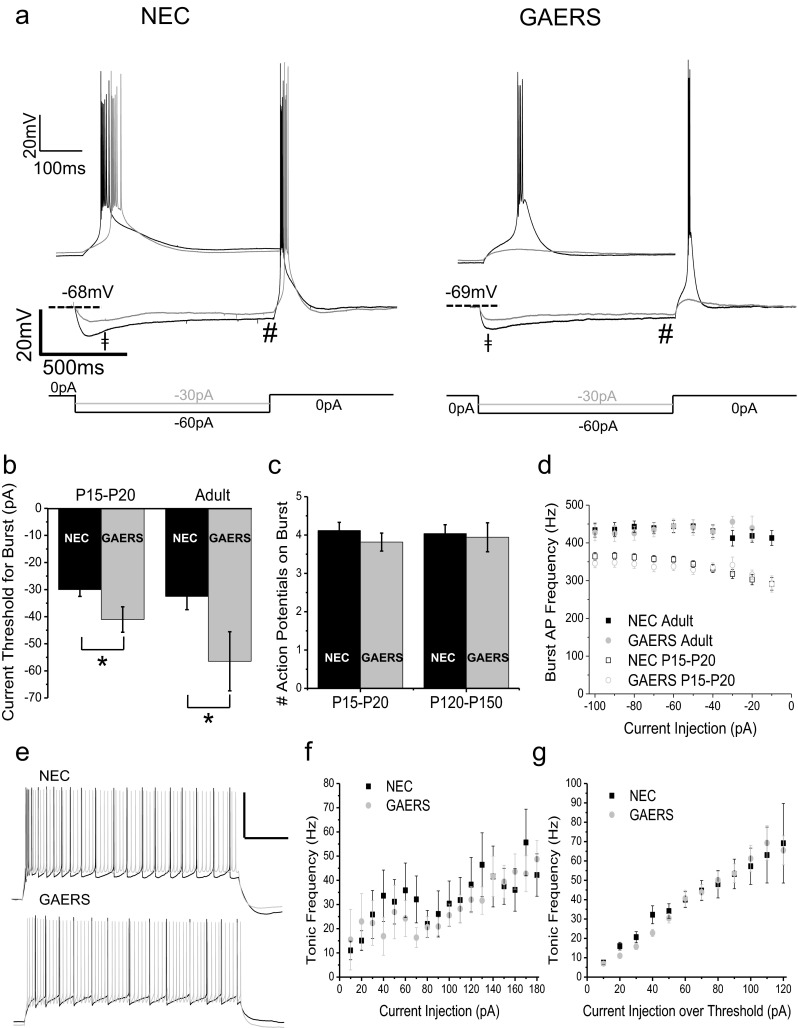
Burst-firing in response to longer duration hyperpolarization. Representative current-clamp recordings from adult NEC (**a**, *left panels*) and GAERS (**a**, *right panels*) VB neurons showing rebound bursts in response to a 1,000-ms hyperpolarizing current injection. The same recordings at a higher time resolution are shown in (**a**, *insets*). **b** Mean data for 1,000-ms current injection required to achieve threshold burst-firing for P15–P20 (NEC (*n* = 61; *black columns*), GAERS (*n* = 39; *grey columns*)) and adult (NEC (*n* = 28; *black columns*), GAERS (*n* = 17; *grey columns*)) VB neurons. **c** The number of action potentials per burst at threshold. **d** Input–output relationships for NEC (*black square symbols*) and GAERS (*grey circle symbols*) VB neurons at both P15–P20 (*open symbols*) and adult (*filled symbols*). **e** Representative current-clamp recordings from P15–P20 NEC (*upper panel*) and GAERS (*lower panel*) VB neurons showing tonic firing in response to a 1,000-ms depolarizing current injection. **f** Mean data for tonic firing frequency in response to increasing current injections and **g** normalized to threshold current for tonic firing in NEC (*n* = 22; *black filled squares*) and GAERS (*n* = 25; *grey open circles*). ǂ = Maximal hyperpolarization, # = Pre-burst membrane potential. *p < 0.05

Current-clamp experiments were initially performed on VB neurons in acute thalamic slices from adult (P120–P150) epileptic GAERS and non-epileptic NEC animals. Individual rebound bursts were generated by 200-ms injections of incrementally decreasing negative current to induce a brief hyperpolarization of the membrane potential, upon cessation of which VB neurons exhibited rebound burst-firing (Fig. 1a, b). Rebound burst-firing in adult GAERS VB neurons required a significantly greater magnitude current injection to achieve threshold compared to adult NEC animals (NEC = −26.9 ± 6.6 pA, GAERS = −48.0 ± 8.0 pA; *p* < 0.01; Fig. 1a right panels, b). In the GAERS model, by 3 months of age, ∼100 % of animals develop an absence epilepsy-like phenotype with characteristic SWDs. Contrastingly, neonatal (P < 30 days) GAERS animals do not display SWD activity [14]. We next examined pre-epileptic P15–P20 NEC versus GAERS VB neurons in order to establish whether the observed attenuated ability to induce burst-firing occurred in conjunction with seizure development. Distinct from that of adult animals, in P15–P20 animals, no significant difference was observed between GAERS and NEC for the amount of current injection required to induce rebound burst-firing in VB neurons with a 200-ms current step (NEC = −45.3 ± 4.1 pA, GAERS = −43.6 ± 7.2 pA; *p* = 0.8; Fig. 1a left panels, b). No differences were observed in the number of action potentials per burst between NEC and GAERS at either the P15–P20 or adult developmental stages (Fig. 1c). Interestingly, burst-firing was observed in many VB neurons in response to depolarizing current, although similar proportions of burst-firing neurons were observed in both NEC and GAERS P15–P20 animals and adults (NEC P15–P20 = 64.9 %, GAERS P15–P20 = 61.1 %; NEC adult = 82.1 %, GAERS adult = 80.0 %). No significant differences were observed in the magnitude of depolarizing current injection required to achieve burst-firing threshold in these neurons (NEC P15–P20 = 38.4 ± 4.8 pA, GAERS P15–P20 = 49.1 ± 7.2 pA; NEC adult = 28.4 ± 4.9 pA GAERS adult = 35.5 ± 9.7 pA). In addition, measurements of resting membrane potential were not significantly different between NEC and GAERS in either P15–P20 or adult VB neurons (Fig. 1e). The input resistance however, as determined from the maximum change in membrane potential upon hyperpolarization during a 1,000-ms current injection divided by the current injected (see Fig. 2a ǂ), was significantly decreased in both adult and P15–P20 GAERS VB neurons (Fig. 1d). Table 1 summarizes the excitability properties associated with rebound burst-firing, and we note that no other significant differences were observed in the various parameters between NEC and GAERS VB neurons. Of note, we also performed current-clamp experiments in P7–P9 NEC and GAERS VB neurons. However, at this developmental stage, we only sporadically observed burst-firing with most neurons generating only one or two action potentials on the crest of a clearly smaller low-threshold spike in comparison to older (P15–P20) animals. This is presumably since the T-type currents at this stage are too small in magnitude to generate robust bursts.

**Table 1:** Burst-firing and passive membrane properties of NEC and GAERS VB neurons

Pre-Epileptic (P15–P20)	Control				ZD7288			
	NEC		GAERS		NEC		GAERS	
n	61		40		8		8	
	Mean	± S.E.	Mean	± S.E.	Mean	± S.E.	Mean	± S.E.
Burst inflection point (mV)	−70.56	0.36	−70.39	0.39	−74.80	1.09	−71.76	1.50
Burst deflection point (mV)	−65.58	2.39	−68.11	1.04	−61.12	2.32	−61.88	1.94
Maximum hyperpolarization (mV)	−82.87	0.71	−82.92	0.83	−95.07	2.62	−96.52	2.00
Pre-Burst Membrane Potential (mV)	−77.88	0.47	−77.10	0.58	−94.68	2.66	−95.50	2.11
Sag (mV)	4.99	0.43	5.81	0.52	0.39	0.35	1.02	0.49
Whole-cell capacitance (pF)	67.02	2.98	71.80	3.09	65.77	8.28	66.00	3.73
Latency to inflection (ms)	65.76	6.07	49.19	6.18	241.93	75.06	236.19	29.45
Latency to AP (ms)	93.00	6.86	73.54	6.92	272.40	76.90	273.73	30.83
Latency inflection to AP (ms)	26.17	1.72	24.34	1.14	30.47	3.48	37.55	7.77
Latency to deflection point (ms)	186.06	11.02	171.30	12.92	377.49	97.19	341.79	34.82
Inflect to deflect latency (ms)	120.29	6.50	122.11	7.76	135.55	31.15	105.60	6.47
Afterhyperpolarization (mV)	−74.83	0.56	−73.71	0.76				
AHP - Vrest (mV)	−1.49	0.28	−0.58	0.43				
Adults (P120–P150)	Control				ZD7288			
	NEC		GAERS		NEC		GAERS	
n	28		17		11		8	
	Mean	±S.E.	Mean	±S.E.	Mean	±S.E.	Mean	±S.E.
Burst inflection point (mV)	−69.34	0.77	−70.30	1.24	−72.99	0.86	−74.17	1.56
Burst deflection point (mV)	−71.32	1.46	−72.32	2.39	−61.99	1.17	−63.60	2.28
Maximum hyperpolarization (mV)	−81.56	0.79	−82.40	1.73	−100.72	1.40	−97.83	2.51
Pre-Burst Membrane Potential (mV)	−77.34	0.70	−78.47	1.42	−99.73	1.42	−95.70	2.73
Sag (mV)	−4.22	0.33	−3.93	0.63	0.99	0.27	2.13	0.50
Whole-cell capacitance (pF)	51.03	3.01	50.18	4.82	48.43	7.23	59.08	4.60
Latency to inflection (ms)	75.53	9.39	62.07	9.80	194.48	21.49	145.46	51.83
Latency to AP (ms)	100.43	9.68	87.34	10.92	224.45	21.87	171.30	53.34
Latency inflection to AP (ms)	24.90	1.17	25.27	1.85	29.97	1.64	25.83	2.21
Latency to deflection point (ms)	261.64	49.66	198.30	21.28	295.72	22.93	249.21	57.41
Inflect to deflect latency (ms)	186.11	49.60	142.07	14.75	101.24	8.73	103.75	8.82
Afterhyperpolarization (mV)	−76.39	0.9562	−75.398	2.1122				
AHP - Vrest (mV)	−2.24	0.6361	−2.6525	0.3943				

### Longer duration hyperpolarization induces rebound bursts with similar thresholds in both adult and P15–P20 GAERS

The response of VB neurons to longer duration (1,000 ms) current pulses was next assessed in order to determine whether allowing more time for endogenous T-type calcium channels to de-inactivate affected rebound bursting ability [6]. Similar to that for 200-ms current injections, adult VB neurons required significantly greater magnitude current injection to reach burst-firing threshold in GAERS compared to NEC animals (NEC = −32.5 ± 4.9 pA, GAERS = −56.5 ± 10.9 pA; *p* < 0.05; Fig. 2a, b). Distinct from that for 200-ms current injections however, in response to a 1,000-ms hyperpolarizing current injection, pre-epileptic P15–P20 VB neurons also required significantly greater magnitude current injection to reach burst-firing threshold compared to NEC (NEC = −30.0 ± 2.5 pA, GAERS = −41.1 ± 2.7 pA; *p* < 0.05; Fig. 2b). This result indicates that the ability to attenuate burst-firing in GAERS VB neurons can precede the development of the SWDs associated with absence seizures.

We further examined the number of action potentials per burst at threshold in response to 1,000-ms current injection and found no differences between adult or P15–P20 NEC and GAERS (Fig. 2c). Similarly, full input–output relationships for action potential frequency during burst-firing did not reveal any significant differences between NEC and GAERS VB neurons at either developmental stage (Fig. 2d). Further, neither the maximum magnitude of membrane hyperpolarization induced by threshold current injection (Fig. 2a ǂ, Table 1), the subsequent I_h_-mediated depolarizing sag of membrane potential, nor the final pre-burst membrane potential on cessation of hyperpolarizing current injection (Fig. 2a #, Table 1) was significantly different between P15–P20 or adult NEC and GAERS VB neurons. As such, the larger injection of current required to achieve the hyperpolarized membrane potential necessary to induce rebound burst-firing may occur as a result of the decreased input resistance selectively observed in GAERS VB neurons (Fig. 1d). No significant differences were observed in the tonic firing properties of NEC and GAERS VB neurons at either P15–P20 or adult stages in response to 1,000-ms depolarizing current injections of increasing magnitude (Fig. 2d–f). Note that tonic firing frequency was analyzed in voltage traces between 500 and 1,000 ms after initiation of current injection in order to avoid any contribution of burst-firing action potentials in response to the depolarizing current injection.

### I_h_ current density is selectively increased in GAERS VB neurons

**Fig. 3:**
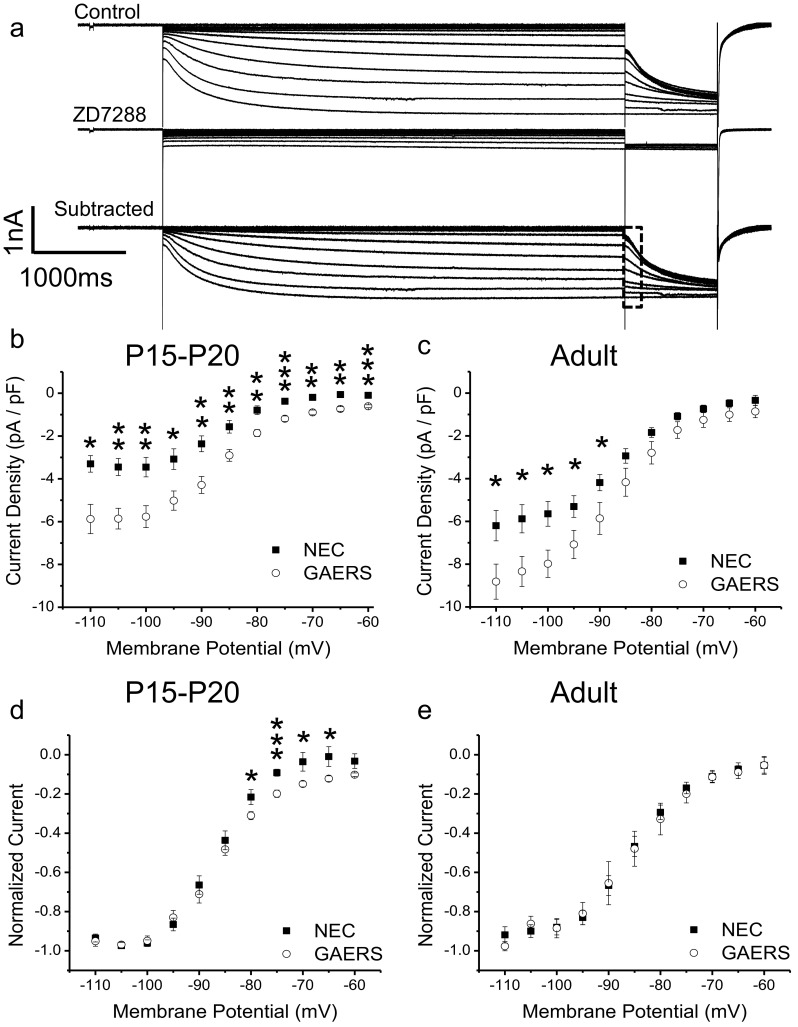
I_h_ current density is increased in GAERS VB neurons. Hyperpolarization-activated current (**a**, *upper panel*), current remaining after ZD7288 (20 μM) (**a**, *middle panel*) and the ZD7288-sensitive (primarily I_h_) component (via subtraction) (**a**, *lower panel*). *Dotted box* shows the portion of tail current trace used in the analysis. Mean data from peak tail currents resulting from a 1-s voltage step to −110 mV from a 5-s voltage holding step (−60 to −110 mV; increment of 10 mV) are shown for P15–P20 (**b** NEC (*n* = 8; *filled squares*), GAERS (*n* = 8; *open circles*)) and adult (**c** NEC (*n* = 13; *filled squares*), GAERS (*n* = 9; *open circles*)) VB neurons. Normalized I_h_ currents (from Fig. 3b, c) are shown for P15–P20 (**d**) and adult (**e**) VB neurons. *p < 0.05, **p < 0.01, ***p < 0.005

A previous study identified an increase in the mRNA expression of the hyperpolarization and cyclic nucleotide-activated (HCN-1) channel isoform in the GAERS VB region using in situ hybridization [27]. This family of voltage-gated ion channels is gated in response to hyperpolarization and cyclic nucleotides and conducts a non-selective cation current which induces membrane depolarization [3]. In order to examine whether the I_h_ current in VB neurons is altered between NEC and GAERS, we measured I_h_ using voltage clamp in acute brain slices. The I_h_ current was recorded using a 250-ms hyperpolarizing voltage step to −110 mV from a variety of holding potentials which were then repeated in the presence of the I_h_ blocker, ZD7288, and subtracted from control to give the ZD7288-sensitive current (Fig. 3a). Analyzing tail currents in the ZD7288-sensitive component showed that I_h_ current density was significantly increased in both P15–P20 and adult GAERS VB neurons compared to NEC (Fig. 3b, c). No significant differences were observed in the voltage dependence of activation of the I_h_ current between adult NEC and GAERS VB neurons (Fig. 3e). P15–P20 GAERS neurons displayed a small but significant apparent increase in the proportion of activated HCN channels at voltages between −80 and −60 mV (Fig. 3d). Despite this, neither the V_50_ for activation (NEC = −86.2 ± 0.9 mV, GAERS = −86.5 ± 2.1 mV) nor slope constant (NEC = 4.2 ± 0.3, GAERS = 5.1 ± 0.3) displayed any significant differences for P15–P20 VB neurons.

### HCN-1 and HCN-3 protein expression is increased in GAERS VB thalamus

**Fig. 4:**
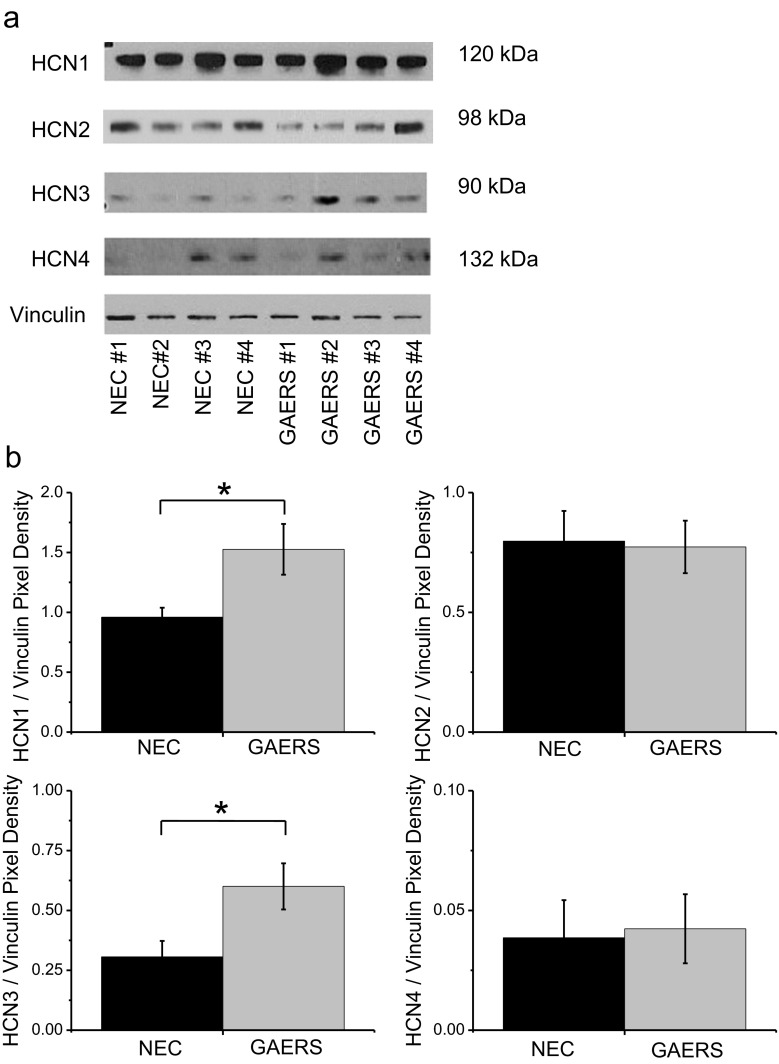
HCN protein isoforms are differentially expressed in GAERS VB thalamus. **a** Representative Western blots for HCN proteins from adult NEC and GAERS VB samples. Primary antibodies to detect each of the HCN channel isoforms were compared to vinculin in order to normalize the expression data. **b** Mean Western blot densitometry data for HCN1–4 normalized to vinculin for NEC (*n* = 12 samples (six animals); *black columns*) and GAERS (*n* = 12 samples (six animals); *grey columns*) VB samples

Given the observed selective increase in I_h_ in GAERS over NEC VB, thalamic tissues were dissected from NEC and GAERS animals and assessed for the levels of HCN isoform proteins. Consistent with the previously noted increase in HCN-1 mRNA [27], Western blotting performed on VB samples revealed a significant increase in the expression of HCN-1 protein (NEC = 0.96 ± 0.08, GAERS = 1.53 ± 0.21; *p* = 0.02) and a further significant increase in the expression of HCN-3 channel protein (NEC = 0.31 ± 0.07, GAERS = 0.60 ± 0.10; *p* = 0.02; Fig. 4a, b). No significant changes were observed in the expression of either HCN-2 or HCN-4 proteins between GAERS and NEC (Fig. 4a, b). Note that comparisons can only be made between NEC and GAERS for a specific isoform since antibody efficiency and exposure time differs between the various HCN isoforms.

### T-type calcium current density is decreased in GAERS VB neurons

The role of T-type calcium channels in the generation of neuronal burst-firing is well documented (for review, see [6]). These low-threshold channels open at more hyperpolarized membrane potentials than other types of calcium channels and uniquely endow them with the ability to generate low-threshold calcium potentials (LTCPs). LTCPs provide an underlying basis for the burst-firing that is commonly observed in the thalamocortical system and, in particular, in TR and RTN neurons. Enhanced thalamic T-type channel mRNA expression [5, 45] and calcium currents [47] have been observed in a number of animal models, including that of GAERS. Further, an underlying genetic difference between the GAERS and NEC strains has been demonstrated to be a gain-of-function mutation in the GAERS Ca_V_3.2 T-type channel [38], although expression of this isoform is restricted to RTN neurons [44].

**Fig. 5:**
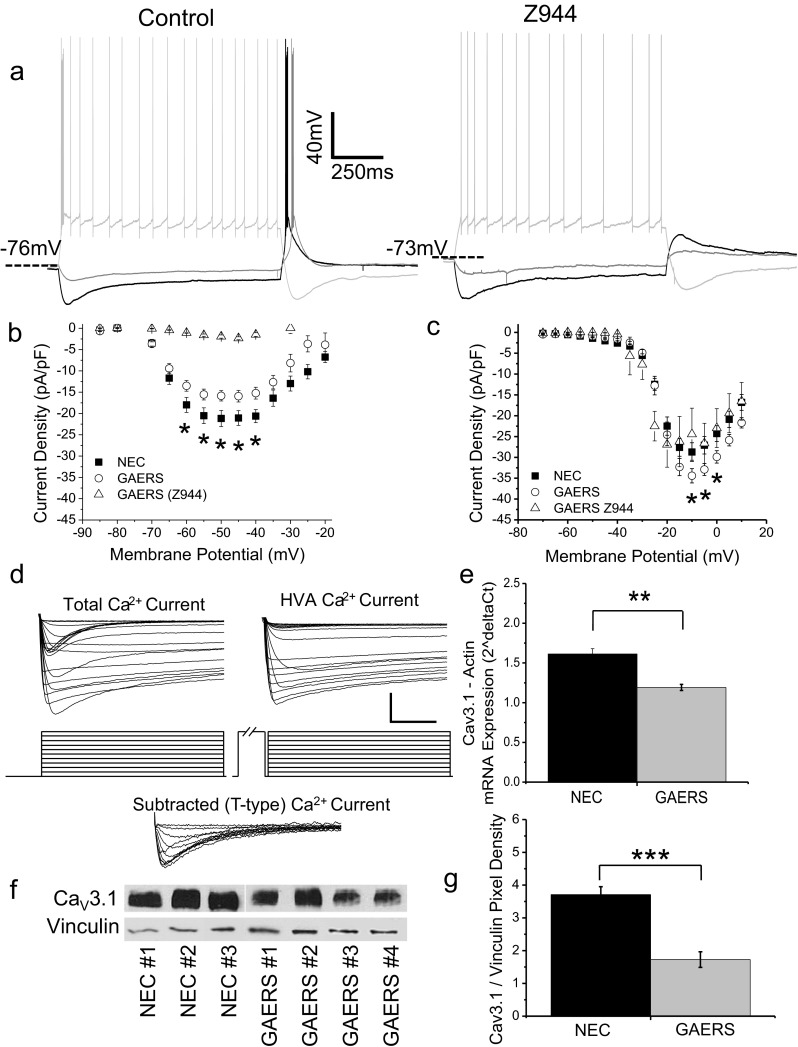
Calcium currents are altered in GAERS VB neurons. Representative trace showing burst-firing in a P15 GAERS VB neuron in response to current injection (**a**, *left panel*) and in the same neuron following application of Z944 (1 μM) (**a**, *right panel*). **b** Whole-cell calcium currents recorded from P7–P9 VB neurons under voltage-clamp conditions. High-voltage-activated calcium currents were isolated by applying a pre-pulse (−50 mV for 100 ms, 10 ms interpulse interval) and subtracted from total calcium currents to yield T-type calcium currents (see “Materials and methods”). Mean current density data for (NEC (*n* = 15; *closed squares*) and GAERS (*n* = 15; *open circles*) is shown for T-type (**b**) and HVA (**c**) calcium currents. Mean current density graphs show GAERS calcium currents in the presence of pan T-type calcium channel blocker, Z944 (1 μM; *n* = 5; (**b)** and (**c)**, *open triangles*). **d** Representative calcium currents recorded from VB neurons in acute brain slices from P7–P9 GAERS. High-voltage-activated calcium currents were recorded by preceding I–V steps with a 100-ms step to −50 mV (**d**, *upper right panels*; 100-ms step shortened in figure to increase timescale). These were subtracted from the total calcium currents acquired from a normal I–V protocol (**d**, *upper left panel*) to yield T-type calcium currents (**d**, *lower panel*). Scale bars = 40 ms and 500 pA. **e** Expression of Ca_V_3.1 mRNA measured by real-time qPCR for samples from VB thalamus for NEC (*n* = 3; *black columns*) and GAERS (*n* = 3; *grey columns*). **f** Western blot of adult VB thalamic samples dissected from NEC and GAERS rats (*upper panels*). Primary antibody to detect Ca_V_3.1 was compared to vinculin in order to normalize expression. Mean Western blot densitometry data normalized to vinculin (*lower panels*) for NEC (*n* = 3; *black columns*) and GAERS (*n* = 4; *grey columns*) VB samples. *p < 0.05, **p < 0.01

Previous experiments have demonstrated that TR neurons are incapable of burst-firing in mice wherein the Ca_V_3.1 T-type channel has been genetically ablated and further that these mice are resistant to pharmacologically induced absence seizures [26]. Given the relationship between T-type calcium channels, burst-firing, and epilepsy [2, 8, 39], we sought to confirm the importance of T-type calcium channels in generating burst-firing in VB neurons. Application of the high-affinity pan T-type calcium channel blocker, Z944 [46] showed that under current-clamp conditions, Z944 (1 μM) completely abolished burst-firing without significantly altering input resistance or resting membrane potential (not shown). This confirms that in the absence of T-type calcium channels, VB neurons are incapable of burst-firing and also that the overshoot induced by the I_h_-mediated rebound in itself is insufficient to generate burst-firing (Fig. 5a).

The current density of calcium currents expressed in NEC vs GAERS VB neurons was also examined under voltage-clamp conditions in acute brain slices. These experiments were restricted to P7–P9 animals in order to achieve the necessary clamping conditions for recording whole-cell calcium currents, which is not feasible in older animals. Calcium currents were elicited by applying incremental (5 mV) voltage steps from a holding of −90 mV. A pre-pulse method (−50 mV for 100 ms) was used to isolate high-voltage-activated (HVA) calcium currents, which could then be subtracted from total calcium currents to identify low-threshold T-type currents (see “Materials and methods”). Interestingly, VB neuron T-type current density was found to be significantly decreased in P7–P9 GAERS compared to NEC at membrane potentials between −60 and −40 mV (NEC = −21.2 ± 1.9, GAERS = −15.9 ± 1.3 pA/pF peak current density; *p* < 0.05, Fig. 5b). Also of note, at membrane potentials between −10 and 0 mV, HVA currents density displayed a modest but significant increase in GAERS compared to NEC neurons (NEC = −28.7 ± 2.2, GAERS = −34.4 ± 1.7 pA/pF peak current density; *p* < 0.05, Fig. 5c). This was confirmed by application of Z944 (1 μM) which completely abolished T-type currents in GAERS P7–P9 VB neurons (−2.3 ± 0.3 pA/pF peak current density, EC_50_ = 3.8 ± 1.5 nM; Fig. 5a, b) without significantly affecting HVA currents (−26.9 ± 5.4 pA/pF, Fig. 5a, c). Expression of Ca_V_3.1 channel-specific mRNA was then evaluated by real-time qPCR in samples of VB thalamus. Ca_V_3.1 mRNA expression was indeed found to be significantly reduced (Fig. 5e; NEC = 1.61 ± 0.07, GAERS = 1.19 ± 0.04; *p* < 0.001) in thalamic samples containing adult VB tissue, but unexpectedly was not significantly reduced in P9 animals (NEC = 0.84 ± 0.17, GAERS = 0.77 ± 0.11; *p* > 0.05, *n* = 3). As expected, the expression of the Ca_V_3.2 and Ca_V_3.3 T-type isoforms was comparatively quite low in VB thalamus, and further, there was no significant difference in the expression of Ca_V_3.2 (NEC = 0.048 ± 0.007, GAERS = 0.047 ± 0.013; *p* > 0.05) or Ca_V_3.3 (NEC = 0.12 ± 0.04, GAERS = 0.09 ± 0.03; *p* > 0.05) mRNAs in VB samples between NEC and GAERS. The expression of Ca_V_3.1 channel protein was confirmed using Western blot of VB tissue wherein a significant reduction was observed in Ca_V_3.1 channel protein levels in adult GAERS compared to NEC (Fig. 5f, g). Western blotting was also performed using Ca_V_3.2 and Ca_V_3.3 antibodies on VB samples from NEC and GAERS; however, protein levels were too low to be accurately detected (not shown).

### Burst-firing in GAERS VB neurons is normalized by I_h_ blockade

**Fig. 6:**
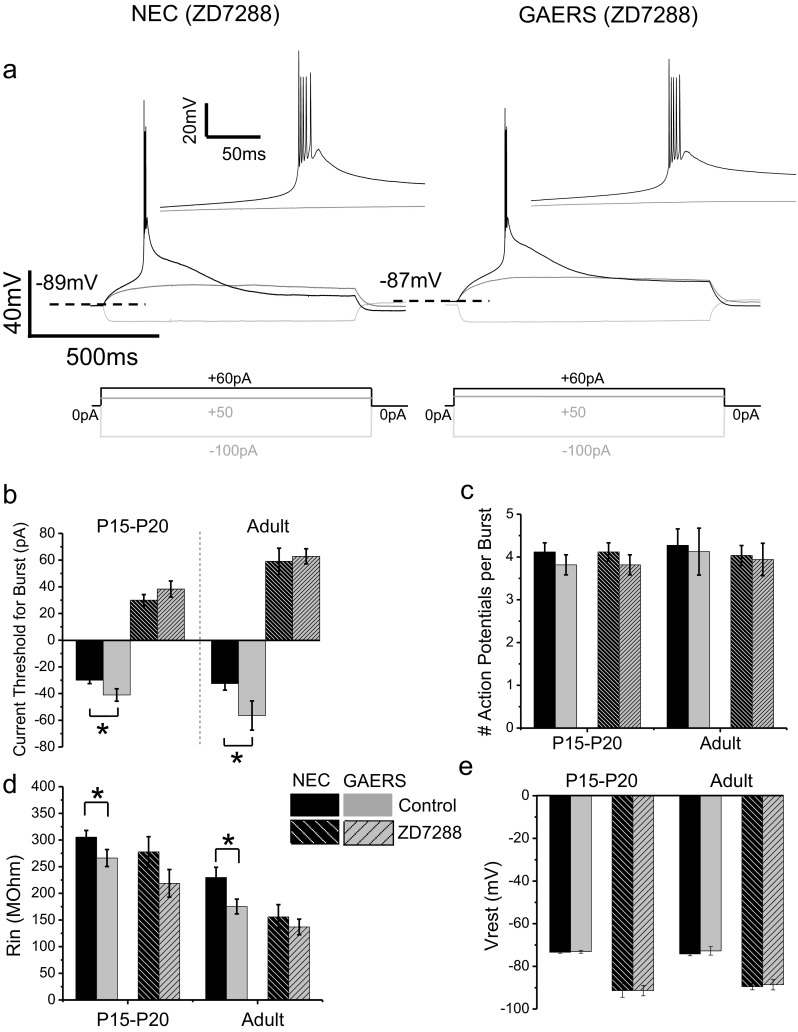
Blockade of I_h_ normalizes VB burst-firing activity. Representative current-clamp recordings from adult NEC (**a**, *left panels*) and GAERS (**a**, *right panels*) VB neurons in the presence of ZD7288 (20 μM). The same recordings at a higher time resolution are shown as insets (**a**, *insets*). **b** Mean data for current required for threshold burst for adult (NEC (*n* = 28; *white-striped black columns*), GAERS (*n* = 17; *black-striped grey columns*)) and P15–P20 (NEC (*n* = 7), GAERS (*n* = 8)) VB neurons. Control data from Fig. 1 is given for comparison for NEC (*solid black columns*) and GAERS (*solid grey columns*). **c** The number of action potentials per burst at threshold. **d** Mean data for input resistance. **e** Resting membrane potential at rest. *p < 0.05, **p < 0.01, ***p < 0.005

Given the enhanced whole-cell I_h_ current observed in GAERS VB neurons, we further evaluated the effect of I_h_ blockade on burst-firing. ZD7288 (20 μM) induced hyperpolarization of the membrane potential (compare Figs. 6a, e and 2a) and decreased the input resistance (Fig. 6d) which is unexpected for blockade of a depolarizing I_h_ conductance basally active at resting membrane potentials. Under these conditions, VB neurons required depolarizing rather than hyperpolarizing current injection to induce burst-firing. This likely occurs as ZD7288-mediated hyperpolarization of the membrane potential de-inactivates T-type calcium channels. Further, the resting membrane potential is no longer depolarized sufficiently to achieve activation of T-type calcium channels upon rebound (Fig. 6a, b). Importantly, in the presence of ZD7288, both P15–P20 and adult GAERS VB neurons no longer required a greater magnitude current injection to burst-fire compared to NEC VB neurons, indicating that I_h_ is indeed responsible for the suppressed burst-firing observed in GAERS (Fig. 6b). No significant differences were observed between NEC and GAERS VB neurons in the number of action potentials per burst (Fig. 6c) or in passive membrane properties such as input resistance and resting membrane potential in the presence of ZD7288 (Fig. 6d, e). To assess burst-firing from the same resting membrane potential as observed in control (pre-ZD7288) conditions, we attempted to restore the resting membrane potential to pre-I_h_ blockade levels using constant depolarizing current injection. However, most neurons would not maintain a steady membrane potential under these conditions due to intrinsic membrane bistability, whereby current injection would instead depolarize neurons to ∼10–20 mV past the control level resting membrane potential or steadily hyperpolarize back to ∼−90 mV [49].

## Discussion

The thalamocortical network is known to be critical for the generation and propagation of absence seizures, although the contributions of individual thalamic regions towards pathophysiological network oscillations remain to be clearly defined. Variability exists across different animal models, which may in fact reflect the wide variety of pathophysiological parameters observed in epilepsy patients and that are defined under the umbrella term of “absence seizures.” Alternatively, the various animal models (some genetic, some chemically/drug-induced) may reflect each particular models specific mechanistic origin. Indeed, in some models, TR neurons have a clearly defined role in the generation of thalamocortical oscillations and contribute to absence seizures [10]. In the naturally occurring GAERS model, TR neurons do not appear to burst-fire during absence seizures [8, 9, 31, 33]. This may reflect that in GAERS, either that the specific region(s) that returns bursting volleys to the cortex has not been recorded from as yet, or that our understanding of absence seizures may not adhere to the current thalamocortical theory in this model. Indeed, with sufficient and self-sustained cortical drive, corticoreticular inhibition may simply induce a silencing of the GAERS TR neurons, preventing sensory relay in a top down fashion, as opposed to cyclical thalamocortical oscillatory manner in the classical theory. Regardless, VB neurons, which project to and receive input from or near the currently accepted cortical origin of SWDs do not respond with bursting in this genetic model [9, 31, 36, 51].

In the current study, we established that GAERS VB neurons display an inherent suppressed ability to burst-fire as they required a significantly greater magnitude of hyperpolarizing current injection in order to generate rebound burst-firing. Concomitant with suppressed burst-firing, we also observed an enhanced basal I_h_ current, consistent with the greater magnitude of required hyperpolarizing current injection. The data are consistent with a previous study indicating that blockade of I_h_ led to an enhancement of rebound burst-firing in non-epileptic Sprague–Dawley and Wistar rats [1]. Experiments in mice VB neurons have shown that blockade of I_h_ with propofol induces a greater time delay from cessation of hyperpolarizing current injection to burst-firing and that both propofol and ZD7288 slow membrane oscillations induced by electrical stimulation of corticothalamic afferents [50]. Therefore, it is feasible that the enhanced I_h_ current in GAERS VB neurons may act to increase the frequency of slow membrane oscillations observed during GAERS SWDs, despite suppressing burst-firing, perhaps due to interplay with T-type calcium window and small-conductance calcium-activated potassium (sK) currents [13]. Interestingly, a recent paper examined the role of large conductance calcium-activated potassium (BK_Ca_) channels on burst-firing properties of dorsolateral lateral geniculate (dlLGN) TR neurons in the WAG/Rij model [17]. It was found that while blockade of BK_Ca_ channels resulted in an increase in the number of action potentials per burst in dlLGN neurons of non-epileptic controls, it had little effect on burst-firing in WAG/Rij animals. Thus, it is likely that valuable information would be gained from investigation of the role of BK_Ca_ and similar ion channels in the GAERS model.

It follows logically that increased I_h_ current density increases the current injection required to burst-fire by increasing membrane conductance and thereby decreasing input resistance. This is supported by the findings that other parameters such as resting membrane potential, the amount of hyperpolarization required for bursting, the resulting membrane potential sag on rebound, and the membrane potential from which the burst initiates are all unaltered between NEC and GAERS VB neurons. In other words, the VB neurons still achieve the same hyperpolarization and burst-fire in the same way; they simply require additional current to initiate the bursting process.

The basal suppression of burst-firing ability likely relates mechanistically to the inability of VB neurons to burst during SWDs in the GAERS model. However, why this occurs in GAERS VB neurons remains unclear since suppressed burst-firing in these neurons would be expected to reduce absence seizures. One possibility is that over generations of breeding GAERS, the VB has developed a hypoexcitable burst-firing behavior as a response to the upstream corticoreticular hyperexcitability. Although, if such a physiological response has evolved as part of an attempted compensatory mechanism of controlling seizures, it has thus far failed in the sense that 100 % of adult GAERS animals exhibit SWDs and seizures. Indeed, given that the suppressed burst-firing phenotype and the enhanced I_h_ current are observed in both adult epileptic and P15–P20 pre-epileptic GAERS, any apparent compensation is unlikely to be directly related to the development of SWDs that present only as the animals age. Also interesting albeit unexplained is that in response to shorter 200-ms current injections (as opposed to 1,000 ms), only adult VB neurons demonstrated suppressed burst-firing, despite both age groups displaying enhanced I_h_ currents.

Blockade of I_h_ with ZD7288 resulted in normalization of burst-firing in NEC vs GAERS VB neurons, despite the fact that the bursts occurred from a more hyperpolarized resting membrane potential and were, therefore, occurring in response to depolarization instead of hyperpolarization. This supports the notion that increased I_h_ current indeed underlies the suppressed burst-firing observed in GAERS VB neurons. This is also supported by a previous study which demonstrated that TR neurons in genetically ablated HCN-2 null mice display a hyperpolarized resting membrane potential and resulting burst-firing in response to depolarizing current injections [28]. Furthermore, these mice display spontaneous absence seizures, presumably due to the fact that depolarizing input from the cortex would be expected to induce burst rather than tonic-firing in these animals. Similarly, another mouse strain displaying a spontaneous mutation in the HCN-2 gene, resulting in an ∼90 % knockdown of HCN-2 mRNA, displays spontaneous absence seizures, as well as occasional tonic–clonic seizures [11]. Of note, while in one report ZD7288 has been suggested to block T-type calcium channels [40], since burst-firing remained robust following the application of ZD7288 (Fig. 6), we do not believe that any such inhibition would have significantly impacted the results. Furthermore, in the presence of the anti-epileptic T-type calcium channel blocker Z944 [46], burst-firing was completely abolished (Fig. 5a) confirming that in the absence of T-type currents burst-firing is not possible.

Western blotting indicted that HCN-3 and HCN-1 protein expression levels are higher in the GAERS VB thalamus than in NEC, supporting our electrophysiological data and also those of a previous report indicating increased HCN-1 mRNA using in situ hybridization [27]. In the previous study, HCN-3 channel mRNA was not assessed. Together, the increased levels of HCN-1 and HCN-3 proteins could explain the enhanced I_h_ current density that we observed. Of interest, Kuisle and colleagues demonstrated that an increase in HCN-1 channel expression correlates with a decreased sensitivity of the I_h_ current to near-physiological application of cAMP. This finding fits well since the HCN-1 isoform is relatively insensitive to cAMP [48]. Given that HCN-3 is also relatively insensitive to cAMP, it follows that this isoform likely contributes to the decreased response of GAERS VB neurons to cAMP observed by Kuisle and colleagues [27, 48]. The sensitivity to cAMP itself has been previously suggested to affect the I_h_-mediated switch from tonic to burst-firing in thalamic neurons, which itself would have a key role in absence seizures [23, 27]. It should also be mentioned that HCN-1, HCN-2, and HCN-4 channels are upregulated in the WAG/Rij model of absence epilepsy, while a decrease in HCN-3 was also observed [23]. In another point of note, we observed a small but significant increase in the proportion of activated I_h_ channels in GAERS compared to NEC P15–P20 VB neurons between −80 and −60 mV (Fig. 3d) and that was not reported in the previous study by Kuisle and colleagues. One possible explanation for this discrepancy is that we were not assessing responses to cAMP and as such were able to utilize ZD7288 to more selectively block I_h_. This allowed us to perform current subtraction to isolate the ZD7288-sensitive current, removing contamination from non-HCN (i.e., leak and inward-rectifying potassium) channels that contribute to the total hyperpolarization-activated current. Another possible explanation to be considered is that the T-type window current is likely active near −60 mV and that ZD7288 has been reported in one study to partially block T-type currents [16, 40]. As such, the increased apparent increase in the activated I_h_ current at these potentials may occur as a result of partial T-type window current blockade contaminating the subtracted ZD7288-sensitive current to a different degree in NEC and GAERS VB neurons. In the previous study, it was reported that no significant differences were observed in the active burst-firing properties of VB neurons between NEC and GAERS [27]. However, the values for threshold current injection required to induce burst-firing were not reported, and those parameters that were reported indicate that the bursts themselves are not significantly altered. This is in agreement with our findings and conclusion that VB neurons still achieve the same hyperpolarization and burst-fire in the same way; they simply require additional current to initiate the bursting process.

In addition to increased I_h_ current density, we also observed a decrease in the current density of T-type currents and a slight increase in the current density of HVA current in GAERS VB neurons. Reduced Ca_V_3.1 mRNA and protein levels in adult VB thalamus both support the electrophysiological findings. We were unable to detect a significant reduction in Ca_V_3.1 mRNA in samples of thalamus taken from P9 GAERS compared to NEC animals. This may result from contamination of the samples with non-VB thalamic regions due to errors in dissection accuracy as a result of the small brain size. The decreased T-type current density might contribute to the suppressed burst-firing in GAERS, although since a non-significant increase was observed for the current injection required to burst-fire between NEC and GAERS in the presence of I_h_ blockade (Fig. 6b), it appears that the small decrease in T-type current density has no significant contribution. T-type calcium currents in GAERS VB neurons have been assessed in a previous study wherein no significant differences were observed at P11–P14 or P15–P17 between NEC and GAERS [47]. This contrasts our findings of reduced T-type calcium current density in GAERS VB neurons. One explanation might be the different methods of preparation as the process of acute dissociation used by Tsakiridou and colleagues would be expected to remove a large proportion of the T-type calcium channels given their well-documented enhanced dendritic expression [22, 29]. In the acute brain slice preparation we utilized, intact neurons were patch clamped wherein dendritic and somatic T-type calcium channels would both contribute to whole-cell currents (although with admittedly poorer space clamp achievable in comparison to acutely dissociated neurons). Another compounding factor might be that our data are normalized to whole-cell capacitance to provide current density, taking into account the fact that larger neurons often have larger currents. In reporting absolute current values, greater error can be introduced with respect to relative expression. Also, contradictory to our results is a study where the authors found an increase in Ca_V_3.1 mRNA in the VB thalamus of GAERS compared to NEC at comparable developmental stages to our study [45]. In situ hybridization was used in this case and which may account for some discrepancy. Finally, another factor to consider is that the strains of GAERS and NEC rats bred at the University of British Columbia, Canada (acquired from the laboratory of Dr Terence O’Brien in Melbourne, Australia) may have diverged in relation to the original strain in Strasbourg, France.

In summary, we find that compared to non-epileptic animals, the VB neurons from GAERS display a suppressed burst-firing ability that we hypothesize underlies why they do not burst-fire during SWDs. Mechanistically, the suppressed burst-firing ability occurs as a result of an increase in the I_h_ current and is detectable at both the P15–P20 and adult stages of development suggesting that it does not occur as an adaptive response to SWDs. What part, if any, VB neurons play in absence seizure propagation thus remains to be elucidated for the GAERS genetic model of absence seizures.
